# Persistence of the Omicron variant of SARS-CoV-2 in Australia: The impact of fluctuating social distancing

**DOI:** 10.1371/journal.pgph.0001427

**Published:** 2023-04-17

**Authors:** Sheryl L. Chang, Quang Dang Nguyen, Alexandra Martiniuk, Vitali Sintchenko, Tania C. Sorrell, Mikhail Prokopenko

**Affiliations:** 1 Centre for Complex Systems, Faculty of Engineering, The University of Sydney, Sydney, NSW, Australia; 2 Sydney Institute for Infectious Diseases, The University of Sydney, Westmead, NSW, Australia; 3 Faculty of Medicine and Health, The University of Sydney, NSW, Australia; 4 Centre for Infectious Diseases and Microbiology – Public Health, Westmead Hospital, Westmead, NSW, Australia; 5 Institute of Clinical Pathology and Medical Research, NSW Health Pathology, Westmead, NSW, Australia; Soongsil University, KOREA, REPUBLIC OF

## Abstract

We modelled emergence and spread of the Omicron variant of SARS-CoV-2 in Australia between December 2021 and June 2022. This pandemic stage exhibited a diverse epidemiological profile with emergence of co-circulating sub-lineages of Omicron, further complicated by differences in social distancing behaviour which varied over time. Our study delineated distinct phases of the Omicron-associated pandemic stage, and retrospectively quantified the adoption of social distancing measures, fluctuating over different time periods in response to the observable incidence dynamics. We also modelled the corresponding disease burden, in terms of hospitalisations, intensive care unit occupancy, and mortality. Supported by good agreement between simulated and actual health data, our study revealed that the nonlinear dynamics observed in the daily incidence and disease burden were determined not only by introduction of sub-lineages of Omicron, but also by the fluctuating adoption of social distancing measures. Our high-resolution model can be used in design and evaluation of public health interventions during future crises.

## Introduction

On 24 November 2021 the B.1.1.529 variant of SARS-CoV-2 was reported to the World Health Organization (WHO) from South Africa, and was soon designated as a variant of concern, subsequently named Omicron [1, 2]. Around the same time, the Omicron variant reached Australia. The first cases attributed to Omicron were, for example, reported by the New South Wales Health Department during the week ending 4 December, 2021 [3]. The rapid spread of the Omicron variant dramatically changed the epidemiological situation in the country, starting a new, fourth, pandemic stage in Australia [4]. This occurred just at the time when the previous, third, wave caused by the Delta (B.1.617.2) variant, had begun to subside, having been controlled to a large extent by a comprehensive mass-vaccination campaign, as well as strict and long lockdowns across several affected states and territories (e.g., New South Wales, Victoria, Queensland, and Australian Capital Territory) [5–8].

A traditional description of a pandemic “wave” assumes that a wave includes both upward and/or downward periods, during which the rising/declining case numbers are substantial [9]. The first three pandemic waves in Australia exhibited well-defined upward and downward periods. The first wave in the nation (March–June 2020) peaked at approximately 500 cases per day, i.e., around 20 daily cases per million [10]. The second wave was mostly confined to the state of Victoria (June–September 2020), peaking at approximately 700 cases per day, i.e., around 30 daily cases per million [11, 12]. The third wave (June–November 2021) peaked at approximately 2,750 daily cases, i.e., around 100 daily cases per million. This peak formed by mid-October 2021, and the incidence stabilised in November between 1,200 and 1,600 daily cases, i.e., between 45 and 65 daily cases per million [6, 13].

**Fig 1:**
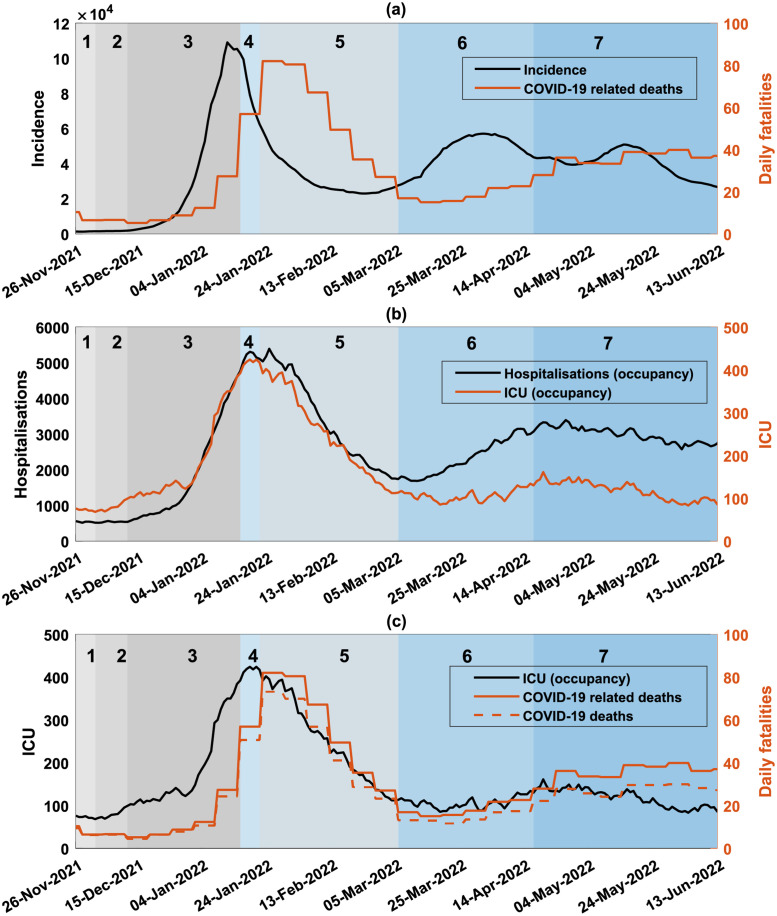
Incidence and disease burden of COVID-19 in Australia between 26th November 2021 and 13th June 2022. Shaded areas in grey and blue show the emergence of variants of concern and sub-lineages over time, identified in weekly genomic surveillance reports (NSW Health). Top (a): Black (y-axis, left): a 7-day moving average of reported COVID-19 daily incidence. Orange (y-axis, right): COVID-19 related deaths. Middle (b): Black (y-axis, left): COVID-19 hospitalisations (bed occupancy). Orange (y-axis, right): COVID-19 ICU cases (bed occupancy). Bottom (c): Black (y-axis, left): COVID-19 ICU cases (bed occupancy); Orange (y-axis, right): Daily COVID-19 related deaths (solid), daily COVID-19 deaths (dashed). The data for COVID-19 cases, hospitalisation and ICU occupancy, and mortality are published by the Australian government.

However, the fourth pandemic stage caused by the Omicron variant (December 2021—September 2022) has included several different waves. Notably, there were multiple peaks, with the first and largest one observed in mid-January 2022 (around 110,000 daily cases, or 4,250 daily cases per million), while smaller but still substantial peaks occurred in early April 2022 (around 57,000 daily cases, or 2,200 daily cases per million) and mid-May 2022 (around 51,000 daily cases, or 2,000 daily cases per million), see Fig 1. The fourth pandemic stage was also characterised by the emergence of a number of sub-lineages of the Omicron variant, some of which rapidly spread across Australia [3]: sub-lineage BA.1 (first reported in NSW by week 48, 2021), BA.2 (first reported in NSW by week 3, 2022), BA.4 (first reported in NSW by week 16, 2022) and BA.5 (first reported in NSW by week 17, 2022). Starting from March 2022, Omicron BA.1 was mostly replaced by BA.2, and from May 2022, BA.4 and BA.5 (see Fig 1). The BA.3 sub-variant did not make a notable impact in Australia.

Different transmission rates were observed between sub-variants: BA.2 had a daily growth rate advantage relative to BA.1 of 0.10 (95% CI: 0.10–0.11) [14] and BA.4 and BA.5 have an estimated daily growth advantage of 0.08 (95% CI: 0.08–0.09) and 0.10 (95% CI: 0.09–0.11), respectively, relative to BA.2 [15]. In addition, BA.4 and BA.5 have resulted in more re-infections than BA.1 and BA.2 [16–18]. Pandemic effects of different Omicron sub-variants have not been traced within an individual-based age-dependent transmission and response model that takes into account demographic heterogeneity of the Australian population.

Age-dependent hospitalisation and fatality rates reportedly differ across all four Omicron sub-variants detected in Australia [3, 19, 20]. In addition, during the Omicron pandemic period, most hospitalisations and deaths occurred among patients aged over 65 years, disabled patients, and those with three or more comorbidities [21]. Thus, to model the actual impact of COVID-19 on mortality and ICU admissions, cases where mortality or critical illness (ICU admission) were directly attributable to the COVID-19 must be distinguished from those where this was not the case. This differential characterisation has has not been modelled for the Omicron pandemic stage in Australia. Hence, the impact of indirect disease burden remains an open question, and providing an adequate model, validated by the actual data from the Omicron pandemic period, may elucidate future public health approaches.

The diverse epidemiological profile of the four co-existing Omicron sub-variants is complicated by fluctuations in the fraction of the Australian population that followed non-pharmaceutical interventions (NPIs), and specifically, social distancing (SD) measures. While during previous pandemic stages the social distancing measures in Australia were mandated by various “stay-in-home-orders”, these restrictions were significantly relaxed during the Omicron pandemic stage. As a result, rather than considering “SD compliance”, we focus on “SD adoption”. Following previous studies [6, 10, 22], we interpret social distancing measures as comprising several behavioural changes that reduce the intensity of interactions among individuals, including staying at home, mask wearing, physical distancing, etc, and thus, reduce the virus transmission probability (see S1 Text, Non-pharmaceutical Interventions). Consequently, the population fraction following one or more of these behaviours, that is, reducing the overall intensity of interactions, is referred to as “SD-adopters”.

Substantial variability in SD-adoption during the Omicron pandemic stage is documented in several reports, including mobility reduction reports, shown in Fig G and Table L in S1 Text. The mobility reduction data are categorised in a coarse-grained way (e.g., workplaces, public transport, residential, retail and recreation, etc.) [23]. Hence, it is difficult to ascertain the extent of actual SD-adoption, i.e., the actual fraction of SD-adopters, at different times during the fourth pandemic stage, especially when interleaved with summer school holidays. It is, however, notable that during the rapid growth in incidence towards the first peak observed in mid-January 2022, the fraction of people staying at home (not travelling to workplaces) increased. Once the incidence significantly dropped during late February 2022, this fraction was reduced. When the incidence resumed its growth toward the second peak in mid-April, there was a slight but delayed increase in this fraction, albeit of a smaller magnitude.

These observations of fluctuating SD-adoption indicate a degree of responsiveness within the population. However, such responsiveness has not yet been quantified and comprehensively modelled, with previous studies assuming only static fractions of individuals compliant with (or adopting) social distancing measures. Thus, there is a need to retrospectively and quantitatively estimate the fluctuating fraction of SD-adopters, treating it as a key variable which determined the pandemic response during the Omicron stage, in presence of other NPIs and vaccination interventions.

## Rationale and objectives

Overall, we aim to provide and validate a high-resolution pandemic response model on the scale of Australia. This model should be able to trace highly transmissible sub-variants of SARS-CoV-2, reproducing and explaining multiple observed waves, evaluate a range of dynamic public health interventions, and quantify their relative contributions to pandemic response.

### Pandemic phases

This study aims to delineate distinct phases of the fourth pandemic stage in Australia, induced by the Omicron variant. In doing so, we separate the dynamics induced by the two initial sub-variants (BA.1 and BA.2), as opposed to the subsequent sub-variants (BA.4 and BA.5). We then model the leading part of the fourth pandemic stage, induced by the two initial sub-variants of Omicron (BA.1 and BA.2), that is, the period between early December of 2021 and mid-April of 2022 (see Fig 1).

### Fluctuating adoption of social distancing

Independently, we also aim to identify the periods of fluctuating adoption of social distancing requirements, matching the corresponding simulations with actual pandemic curves. In identifying the impact of the social distancing behaviour on the pandemic spread, we contrast the trajectory modelled for the fluctuating SD-adoption with its static counterparts, set at feasible extremes of SD-adoption (20% and 70%). In addition, we consider transition to the phase dominated by sub-variants BA.4 and BA.5, and possible reasons behind persistence of the Omicron variant of SARS-CoV-2 in Australia.

### Disease burden

Finally, we model the disease burden of the fourth pandemic stage in Australia experienced until mid-April 2022, in terms of hospitalisations, intensive care units (ICU) occupancy, and mortality caused by the Omicron variant (BA.1 and BA.2). In doing so, we account for the impacts directly attributable to, or not directly attributable to COVID-19 on these parameters. This analysis considers a balance of the fluctuating extent of SD-adoption, transitions across the sub-variants, and the indirect effects of COVID-19, and explores to what extent this balance shaped the nonlinear dynamics across the three measures of disease burden.

## Materials and methods

In order to capture the heterogeneous population structure, we used a high-resolution Agent-based Model (ABM) which comprised an artificial population of over 23.4 million agents generated from the Australian census data. The model was implemented within a large-scale software simulator (AMTraC-19: Agent-based Model of Transmission and Control of the COVID-19 pandemic in Australia [24]) and included a range of non-pharmaceutical interventions and vaccination rollout schemes. This model has been previously calibrated and validated to reproduce key variant-specific epidemiological features of the COVID-19, including the ancestral variant [10, 22] and the Delta variant [6, 25]. In this work, we calibrated the model to the Omicron variant (BA.1 and BA.2), using genomic surveillance reports [3]; extended the social distancing model to support a fluctuating fraction of SD-adopters; carried out an additional sensitivity analysis; and validated the model using the incidence data reported between December 2021 and April 2022, as described in S1 Text.

Agents are grouped in various mixing contexts which reflect the demographic features captured by the census. While contacts in “residential” contexts (household, household cluster, community) correspond to local interactions, other interactions occur in “workplace/education” mixing contexts (working groups and schools). An agent is initialised with a commuting pattern between a residential area and a destination zone representing workplace, using the datasets maintained by the Australian Bureau of Statistics (ABS) [26–28]. The disease transmission probabilities are set to vary across the interaction contexts and ages, see Table B in S1 Text. The transmission is simulated in discrete time, with two steps per day: “daytime” capturing the interactions in workplace/education contexts, and “nighttime” capturing the interactions in residential contexts.

The model implements a specific profile for the natural history of the disease, progressing over several states of infection, i.e., Susceptible, Infectious (Asymptomatic or Symptomatic), and Removed (recovered or deceased). This dynamic is governed by several parameters, including the incubation period and recovery period, which are randomised across the agents around mean values. Susceptible hosts or agents which share a mixing context with an infectious agent may become infected and then infectious. Some agents develop asymptomatic infection, with this fraction set differently for adults and children. The asymptomatic infectivity is set lower than the symptomatic one. The model also specifies case detection probabilities which differ for symptomatic and asymptomatic/pre-symptomatic cases, see S1 Text, Agent-based model and transmission probabilities. Sensitivity analysis is described in S1 Text, Calibration and sensitivity analysis.

A pandemic scenario is triggered by infecting a number of agents residing in metropolitan areas around international airports, in proportion to the number of incoming passengers [6, 10], as described in S1 Text, Agent-based model and transmission probabilities. At each simulation step, the mixing contexts of agents and their trajectory through the natural history of the disease determine their transmission probabilities. Once an agent is exposed to infection, the transmission probability varies: it exponentially grows to a peak of infectivity and then linearly declines during the agent’s recovery.

In modelling the fourth pandemic stage, we assumed that the population vaccination coverage was high. This assumption is supported by the vaccination levels achieved in Australia by the end of 2021. The proportion of adult Australians (i.e., over the age of 16) who were double vaccinated by 30 November 2021 reached 87.2%, and 92.5% of adults have had by then at least one dose [29]. The implemented vaccination scheme follows a pre-emptive mass-vaccination campaign with two vaccines: priority and general (see S1 Text, Vaccination modelling). This reflects key features of the actual vaccine rollout in Australia during 2021 [6, 22]. When an agent is vaccinated, their vaccination state is defined by a set of the corresponding vaccine efficacy parameters.

The model includes several NPIs, including case isolation, home quarantine, school closures, and social distancing behaviour [6, 10]. Case isolation and home quarantine are activated from the start of simulation. The setup and duration of school closures approximates the period of summer holidays and a gradual resumption of school activities until mid-March of 2022. The extent of social distancing adoption is simulated dynamically, with the SD-adopting population fraction set at any given simulation step according to a predefined assignment profile, see Table E in S1 Text. Whenever the SD-adoption fraction changes during the simulation, Bernoulli sampling determines the agents which are selected to follow the social distancing behaviour. Vaccination states, the adoption of social distancing and the compliance with other NPIs modify the transmission probabilities within the agents’ mixing contexts, as described in S1 Text, Non-pharmaceutical Interventions.

Estimation of disease burden is carried out in terms of hospitalisation, ICU occupancy and mortality (daily and cumulative deaths). In doing so, we used age-dependent case hospitalisation risks (CHRs) and ICU ratios, scaled to the actual hospitalisation cases in Australia, and age-dependent infection fatality rates (IFRs), while accounting for adjusted vaccine efficacies and a differentiation between sub-variants BA.1 and BA.2, as described in S1 Text, Modelling disease burden and Mortality statistics.

## Results

### Pandemic phases

**Fig 2:**
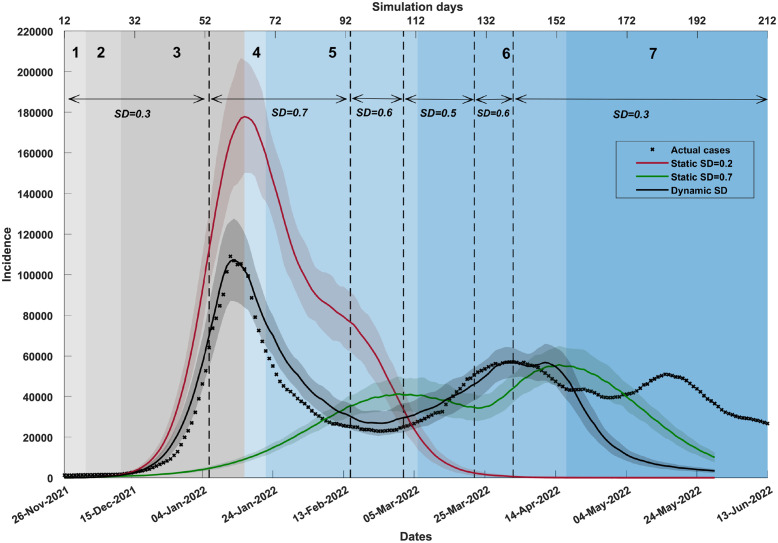
A comparison of incidence produced by different social distancing (SD) profiles. The plot contrasts dynamic social distancing (SD) adoption (shown in solid black) and static SD-adoption fractions (*SD*_1_ = 0.2, shown in red; *SD*_2_ = 0.7, shown in green). Coloured shaded areas around the solid line show standard deviation. Changes in dynamic SD-adoption are marked by vertical dashed black lines. Traces corresponding to each simulated scenario are computed as the average over 20 runs. Simulated SD adoption is combined with other interventions (i.e., school closures, case isolation, and home quarantine). A 7-day moving average of the actual time series (black crosses) is shown for the period between 26th November 2021 and 13th June 2022 (x-axis, bottom). The simulated incidence is scaled up by 10% to reflect the population increase from 23.4 million (2016 census data, model input) to 25.8 million (2021 census data). The simulated incidence is offset by 12 days (x-axis, top) to align with the observed incidence peak. Shaded areas in grey and blue show the emergence of variants of concern and sub-lineages over time, identified in weekly genomic surveillance reports (NSW Health).

Using contemporary genomic surveillance reports [3], we identified seven main phases of the fourth pandemic stage in Australia caused by the Omicron variant, shown in Figs 1 and 2. The timeline is divided into 7 phases as follows:

- Phase 1: BA.1 detected, epidemiological week 48, ending 4 December, 2021.
- Phase 2: Delta and BA.1 co-exist, epidemiological week 49, ending 11 December, 2021.
- Phase 3: BA.1 dominant, epidemiological week 50 (2021), ending 18 December, 2021, to week 2 (2022).
- Phase 4: BA.2 detected, epidemiological week 3, ending 22 January, 2022.
- Phase 5: BA.1 and BA.2 co-exist, epidemiological week 4 to week 9, ending 5 March, 2022.
- Phase 6: BA.2 dominant, epidemiological week 10, ending 12 March, 2022.
- Phase 7: BA.2, BA.4 and BA.5 co-exist, epidemiological week 16, ending 23 April, 2022, onwards Data source: COVID-19 weekly surveillance reports, published by NSW Health [3].

We note that sub-variant BA.1 has displaced the Delta variant by phase 3, causing a rapid growth in incidence towards the primary incidence peak. The second incidence peak shaped during phase 6 which was dominated by sub-variant BA.2. Phase 7 marked the emergence of BA.4 and BA.5.

We re-calibrated the ABM to the incidence data observed during the beginning of the fourth pandemic stage in Australia, driven by the Omicron variant (specifically, phase 3 in Fig 1 shaped by BA.1, but considering also phases 4 to 6 when BA.1 was concurrent with BA.2). For the previous pandemic stage in Australia (June–November 2021), i.e., the third pandemic wave shaped by the Delta variant, the reproductive number was calibrated to be around *R*_0_ = 6.20 [6]. In this study, the calibration resulted in *R*_0_ = 19.56 (see S1 Text, including Calibration and sensitivity analysis and Table C in S1 Text), which is consistent with other reports estimating *R*_0_ of Omicron BA.1 to be approximately 3.1 times as high as the reproductive number of the Delta variant [30].

### Fluctuating adoption of social distancing

In modelling the first six phases during which the two initial sub-variants of Omicron (BA.1 and BA.2) emerged and reached dominance, we focused on a “retrodictive” estimation of the SD-adopting population fraction. A key assumption that we made is that the adoption of social distancing behaviour fluctuates over time and the changes in the extent of SD-adoption are not necessarily synchronous with the pandemic phases. Instead, these changes can be driven by individual perception of the infection risks, life pattern changes associated with summer holidays and return to school/work for many individuals, vaccination availability for children, and other policy setting changes affecting social distancing. Hence the changes in the extent of SD-adoption may precede or follow some salient observed features of the pandemic trajectory.

Selection of the SD-adoption change-points and the corresponding fractions of SD-adopters is a subject of modelling which aims to minimise the difference between the resulting and observed incidence curves. For example, the SD-adoption profile presented in Table E in S1 Text includes six change-points, including the initial assignment, at day 0, of *SD* = 0.3, that is, 30% of the agents follow social distancing behavior from the pandemic stage onset. This is set to continue until day 54 after which the fraction of SD-adopters sharply rises to 70%, i.e., *SD* = 0.7 from day 55, and so on. This optimised profile produced the average incidence curve shown in Fig 2, closely approximating the observed incidence curve, with both primary and secondary peaks well-aligned with the observations. The optimisation process is described in S1 Text, Non-pharmaceutical Interventions.

The incidence curve produced by the fluctuating (dynamic) SD-adoption is contrasted with two static SD-adoption alternatives, set at 20% and 70%, i.e., *SD*_1_ = 0.2 and *SD*_2_ = 0.7. This comparative analysis, illustrated by Fig 2, highlights the nonlinear nature of resultant dynamics: the fluctuating fraction of SD-adopters produces the trajectory which agrees with the actual incidence dynamics, unlike the static SD-adoption profiles which both fail to match the salient pandemic patterns. For example, the lower static SD-adoption, *SD*_1_ = 0.2, does not produce the secondary peak at all, and the higher static SD-adoption, *SD*_2_ = 0.7, misses the first peak by almost two months and produces the second peak with a larger magnitude than the first one (see Table M in S1 Text).

There was a notable divergence between the simulated and observed curves, which developed at the onset of phase 7, in early May 2022 (Fig 1). The incidence observed in phase 7 correlated with the spread of sub-variants BA.4 and BA.5, and the daily cases reached substantial levels, generating a third peak at the end of May. In contrast, the simulated curve fell during this period to near-zero levels. We note that no extent of the SD-adoption fraction simulated between *SD*_*min*_ = 0 and *SD*_*max*_ = 0.7, was sufficient to maintain the infection spread. This can be explained by depletion of the susceptible host population within the simulation: the simulated cumulative incidence at the end of phase 6 reached 23% of the simulated population, with the rest being probabilistically protected by vaccinations (given the partial efficacy of both vaccines). Since the model does not assume re-infections and does not simulate diminishing vaccine efficacy, this divergence strongly suggests that the actual incidence observed during phase 7, associated with the spread of BA.4 and BA.5 (including the third peak, see Fig 1), was mostly generated by re-infections or new infections in vaccinated individuals with lower vaccine effectiveness (e.g., due to waning vaccine efficacy), rather than infections of the epidemiologically or immunologically “naïve” population (see Discussion).

### Disease burden

We next modelled the disease burden generated by the Omicron variant (BA.1 and BA.2), measured in terms of (i) hospitalisations (occupancy), (ii) ICU (occupancy), and (iii) mortality (daily and cumulative deaths) in Australia, as described in S1 Text, Modelling disease burden and Mortality statistics. The key input to the disease burden estimation is provided by the incidence curves produced by the simulation. In other words, a given SD-adoption profile generates a non-linear incidence trajectory which in turn results in hospitalisations and ICU occupancy. The incidence trajectory also leads to estimation of daily and cumulative deaths. Similarly to the analysis of daily incidence, we contrast dynamic and static SD adoption. The difference, however, is that we no longer optimise the SD assignment profile; instead, we use the optimised profile presented in Table E in S1 Text, Modelling disease burden. Thus, a comparison between the actual and estimated disease burdens can be used to further validate the model.

**Fig 3:**
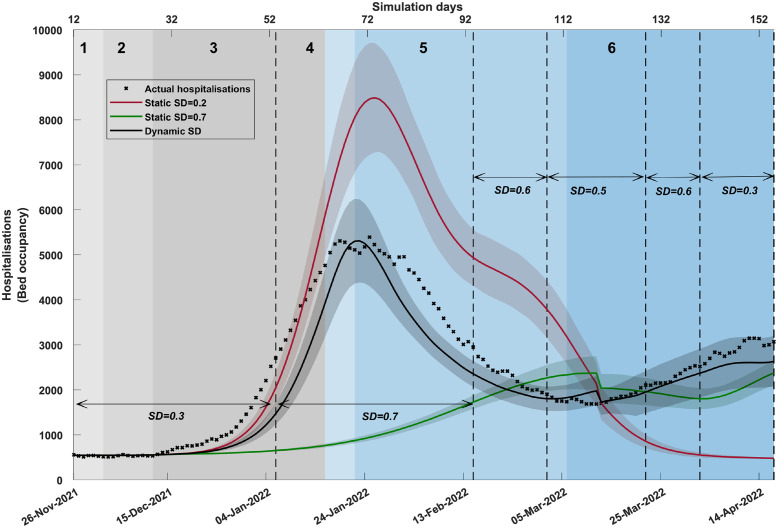
A comparison of hospitalisations (bed occupancy) produced by different social distancing (SD) profiles. The plot contrasts dynamic social distancing (SD) levels (shown in solid black) and static SD levels (*SD*_1_ = 0.2, shown in red; *SD*_2_ = 0.7, shown in green). The simulated hospitalisations are offset by 7 days. Coloured shaded areas around the solid line show standard deviation. Changes in dynamic SD-adoption are marked by vertical dashed black lines. Traces corresponding to each simulated scenario are computed as the average over 20 runs. SD adoption is combined with other interventions (i.e., school closures, case isolation, and home quarantine). The actual time series (black crosses), shown from 26th November 2021, aligns with the start of the Omicron outbreak in Australia. Shaded areas in grey and blue show the emergence of variants of concern and sub-lineages over time, identified in weekly genomic surveillance reports (NSW Health).

Fig 3 and Table N in S1 Text show that the hospitalisation curve (occupancy) generated by the dynamic SD-adoption closely matches the actual hospitalisations. This alignment is again in stark contrast with the hospitalisation curves produced by static SD-adoption fractions (*SD*_1_ = 0.2 and *SD*_2_ = 0.7).

**Fig 4:**
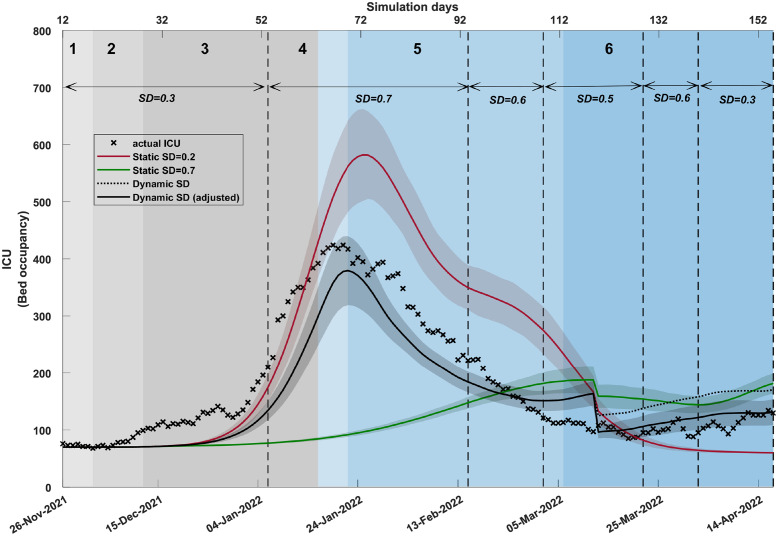
A comparison of ICU (bed occupancy) produced by different social distancing (SD) profiles. The plot contrasts (i) dynamic SD levels, adjusted for phase 6 by scaling with 0.77 to capture ICU cases due to COVID-19 (solid black), (ii) non-adjusted dynamic SD levels to represent all ICU cases (dotted black), and (iii) static SD levels (*SD*_1_ = 0.2, shown in red; *SD*_2_ = 0.7, shown in green). The simulated ICUs are offset by 7 days. Coloured shaded areas around the solid line show standard deviation. Changes in dynamic SD-adoption are marked by vertical dashed black lines. Traces corresponding to each simulated scenario are computed as the average over 20 runs. SD adoption is combined with other interventions (i.e., school closures, case isolation, and home quarantine). The actual time series (black crosses), shown from 26th November 2021, aligns with the start of the Omicron outbreak in Australia. Shaded areas in grey and blue show the emergence of variants of concern and sub-lineages over time, identified in weekly genomic surveillance reports (NSW Health).

Fig 4 and Table N in S1 Text show that the ICU curve (occupancy) generated by the dynamic SD-adoption matches the actual ICU occupancy reasonably well during the initial five phases which were shaped mostly by the BA.1 sub-variant, and somewhat diverges during phase 6 dominated by BA.2. During phase 6 (that is, for the last six weeks of the considered timeline), the simulated ICU occupancy is higher than the actual ICU occupancy. The ICU occupancy model, driven by ICU admission rates, accounted for a reduction in the disease severity, differentiating between BA.1 and BA.2 (cf. Table H in S1 Text). Furthermore, we adjusted the ICU trajectory during phase 6 to represent the ICU occupancy directly attributable to COVID-19, rather than the ICU occupancy in patients with COVID-19 (as explained in S1 Text, Mortality statistics). The adjusted ICU occupancy is in a better concordance with the actual data. The static SD-adoption fractions (*SD*_1_ = 0.2 and *SD*_2_ = 0.7) do not capture the actual trajectory of ICU occupancy.

**Fig 5:**
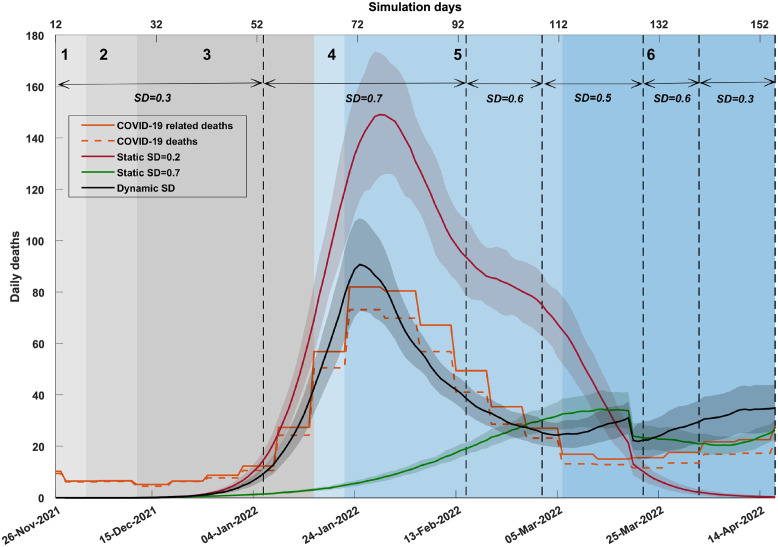
A comparison of daily deaths produced by different social distancing (SD) profiles. The plot contrasts dynamic social distancing (SD) levels (shown in solid black) and static SD levels (*SD*_1_ = 0.2, shown in red; *SD*_2_ = 0.7, shown in green). The simulated daily deaths are offset by 14 days. Coloured shaded areas around the solid line show standard deviation. Changes in dynamic SD-adoption are marked by vertical dashed black lines. Traces corresponding to each simulated scenario are computed as the average over 20 runs. SD adoption is combined with other interventions (i.e., school closures, case isolation, and home quarantine). Actual daily deaths are derived from reported weekly deaths (shown in orange; solid: COVID-19 related deaths; dashed: COVID-19 deaths). Shaded areas in grey and blue show the emergence of variants of concern and sub-lineages over time, identified in weekly genomic surveillance reports (NSW Health). The timeline is divided into 6 phases as follows: 1) BA.1 detected, 2) Delta and BA.1 co-exist, 3) BA.1 dominant, 4) BA.2 detected, 5) BA.1 and BA.2 co-exist, and 6) BA.2 dominant.

Finally, Fig 5, Fig B and Table N in S1 Text show that the daily mortality curve produced by the dynamic SD-adoption matches the actual data reasonably well again. In contrast, the mortality estimated by static SD-adoption fractions, *SD*_1_ = 0.2 and *SD*_2_ = 0.7, consistently overestimate and underestimate the actual observations, respectively.

These estimations of the disease burden reinforce the argument that the nonlinear dynamics of the observed hospitalisations, ICU occupancy, and mortality result from a nuanced combination of (i) fluctuating fractions of SD-adoption, (ii) a transition from BA.1 to BA.2, and (iii) indirect effects of COVID-19, i.e., the distinction between ICU admissions or deaths arising due to COVID-19 versus those with COVID-19.

## Discussion

Emergence of recurrent epidemic waves of COVID-19 is a systemic phenomenon and its quantitative understanding remains an elusive target [31]. Typically, several interacting factors contribute to recurrent dynamics. Firstly, the population structure in the modern world is highly heterogeneous: not only at the level of individual households and local government areas, but also at the regional, national and international level. This heterogeneity forces the infection transmission to follow complex paths, producing distinct urban and rural waves [26, 32, 33], disproportionate effects on high- and low-density housing [34], as well as in the areas characterised by socioeconomic disadvantage profiles and higher concentrations of essential workers [6, 35–37]. Thus, while infection rates may decline in some areas, they may escalate in others.

Secondly, the population compliance with various stay-at-home orders and adoption of social distancing requirements fluctuates in time: strong initial adherence is typically replaced by sizable fatigue and rarely recovers beyond moderate compliance, thus reducing effectiveness of NPIs [6, 38, 39]. This fatigue cannot be exclusively attributed to “contrarian” individuals and affects broad population groups.

Thirdly, vaccine efficacy wanes over time, requiring booster vaccine doses, which temporarily reestablish protective immunity. Independently, following a typically strong initial vaccination uptake, “the fully up to date” vaccinated population fraction slowly reduces over time, undergoing possible upswings when the risk perception increases [40, 41]. Similar to the NPI fatigue, this dynamic is driven not only by “anti-vaxxer” groups, but may depend on access to and knowledge about vaccination [42], and involve several drivers of vaccine hesitancy, including confidence, complacency, altered risk calculation, and limited collective responsibility [43, 44].

In addition, the testing, tracing, isolation and quarantine (TTIQ) capacities themselves may be under stress during rapidly growing outbreaks, diminishing their role in curbing the epidemic. The TTIQ capacities were severely strained in Australia during the rise of the Omicron variant, leading to a decline in their effectiveness [45]. Moreover, delays in imposing strict NPIs or initiating vaccination rollouts amplify non-linearly, so that short response lags cause long recovery tails [6, 10]. All these cyclical spatiotemporal dynamics may combine over time, creating vicious feedback loops and superposition of interacting waves.

In this study, we sought to increase our understanding of the mechanisms generating multiple pandemic waves. We specifically modelled a pandemic response to the emergence and spread of highly transmissible variant of SARS-CoV-2 (Omicron and its sub-lineages) during the fourth pandemic stage in Australia. This stage produced several local incidence peaks, and we identified the corresponding phases (see Fig 1), followed by retrospective modelling of dynamic social distancing behaviour.

Importantly, the identification of distinct phases did not reduce the analysis to a description of well-defined waves, but rather attributed the incidence peaks and patterns to nuanced combinations of new sub-variants and fluctuations in social distancing behaviour. For example, the secondary peak in mid-April of 2022 (see phase 6 in Fig 1) was explained not only by the emergence of BA.2 which started to dominate the preceding BA.1, but also by the reduced adoption of social distancing requirements.

The main result of this modelling is that the fraction of SD-adopters was found to be fluctuating in response to the incidence dynamics. Essentially, these changes appeared in response to the pandemic dynamics observed by the population, sometimes lagging behind and occasionally preempting pandemic turns.

Using the actual incidence data and simulated scenarios which varied the extent of SD-adoption across time, we “retrodictively” determined the dynamically fluctuating SD-adoption profile that produced the incidence curves matching the observations. The resultant incidence curves were further contrasted with the curves produced by alternative scenarios defined by static SD-adoption. The comparison clearly demonstrated that, unlike the dynamic SD-adoption, the static alternatives failed to reproduce key non-linear features of the incidence trajectories. This indicated that the fraction of individuals adopting (or complying with) social distancing requirements may greatly vary over time, especially during a long pandemic. Hence, public health policies must adequately account for this variability, acknowledging and addressing possible fatigue and complacency within the population. Timely, rapidly designed and focused communication campaigns may be needed to reinforce the importance of continuing social distancing in controlling persistent spread and reducing the public health burden.

The study also suggested, albeit indirectly, that the increased incidence observed during the last wave induced by sub-variants BA.4 and BA.5 (Fig 1: phase 7) was mostly driven by reinfections or new infections in vaccinated individuals with low or waning vaccine effectiveness, rather than new infections of the epidemiologically or immunologically “naïve” population. Testing for antibodies in Australia between 9 and 18 June 2022 provided an indication of the extent of cumulative exposure in the community to vaccination and/or natural infection: the prevalence of anti-spike antibodies, elicited by both vaccination and natural infection, was found to be very high (99%) across all jurisdictions, while the prevalence of anti-nucleocapsid antibodies, elicited by natural infection, reached 46% [46]. The finding of our study, which emphasised re-infections and low or diminishing vaccine efficacy as key factors during phase 7, pointed to the need for agile “booster” vaccination campaigns, to raise immunity levels within the population during protracted pandemic stages.

We estimated the corresponding disease burden (the hospitalisations, ICU occupancy, and daily and cumulative deaths), and compared it with the actual data. A good agreement between the simulated and actual disease burden data reinforced the point that the observed nonlinear dynamics are produced not simply by transitions across sub-variants of Omicron, but also in response to varying adoption of social distancing behaviour. The analysis also quantified and emphasised indirect effects of COVID-19 on the disease burden in Australia, differentiating between ICU cases directly attributable to COVID-19 versus those occurring with COVID-19. This distinction highlighted the need for a precise attribution of underlying conditions associated with ICU admissions during a long pandemic.

Several limitations of the study have to be acknowledged. Firstly, the simulated population was generated using the latest available Australian Census data from 2016 (23.4M individuals) and may not be precisely representative of 2021–2022 period of time. However, the generated curves were scaled by 10% to reflect the current Australian population (25.8M). Secondly, the ABM does not simulate transmissions within healthcare facilities. However, health care professionals were vaccinated over several priority phases carried out in Australia in 2021 and 2022 and mask mandates were in place in health care facilities in these time periods, reducing the effect of this limitation. Furthermore, while vaccine efficacy is known to diminish over time, we did not model this explicitly, and considered this effect to be balanced by third vaccination doses administered in Australia during the studied period (e.g., 71.6% of Australian adults had received 3 doses by August 2022). In contrast to the greatly fluctuating SD-adoption, a decline in the vaccine efficacy is known to be monotonic [47], while our focus was on modelling multiple peaks of distinct waves within a single pandemic stage.

In conclusion, the understanding of the impact of the Omicron variant on the COVID-19 pandemic continues to be refined, and our results may benefit from additional analysis of the latest Omicron sub-variants. Nevertheless, the quantitative retrospective modelling presented in this study highlighted the impact of fluctuating social distancing behaviour on the resultant pandemic dynamics, revealing nuanced reasons for persistence of the Omicron variant in Australia. The developed model, validated with actual data from the Omicron stage in Australia, captures multiple dynamic factors which non-linearly affect the pandemic spread, and hence, can be used to evaluate and contribute to agile, adaptive and multi-faceted public health responses in the future.

## Supporting information

S1 Text (PDF)
