# Supplementary material for: Persistence of the Omicron variant of SARS-CoV-2 in Australia: The impact of fluctuating social distancing
Source: PLOS Glob Public Health. 2023 Apr 17;3(4):e0001427. doi: 10.1371/journal.pgph.0001427 (PMC10109475; doi:10.1371/journal.pgph.0001427)
Supplement: S1 Text — (PDF) [file pgph.0001427.s001.pdf]

Persistence of the Omicron variant of SARS-CoV-2 in Australia: The impact of fluctuating social distancing

Sheryl L. Chang [1,2*], Quang Dang Nguyen [1], Alexandra Martiniuk [3], Vitali Sintchenko [2,3,4,5], Tania C. Sorrell[2,3], Mikhail Prokopenko[1,2],

1 Centre for Complex Systems, Faculty of Engineering, The University of Sydney, Sydney, NSW 2006, Australia
2 Sydney Institute for Infectious Diseases, The University of Sydney, Westmead, NSW 2145, Australia
3 Faculty of Medicine and Health, The University of Sydney, NSW, 2006, Australia
4 Centre for Infectious Diseases and Microbiology – Public Health, Westmead Hospital, Westmead, NSW 2145, Australia
5 Institute of Clinical Pathology and Medical Research, NSW Health Pathology, Westmead, NSW 2145, Australia

* sheryl.chang@sydney.edu.au

Supporting information

S1 Text.

Agent-based model and transmission probabilities. In simulating the transmission and control of the COVID-19 pandemic on the scale of Australia, we follow the agent-based modeling (ABM) approach, with more than 23.4M agents generated using data from Australian Census and other data provided by the Australian Bureau of Statistics (ABS) and the Australian Curriculum and Assessment and Reporting Authority (ACARA) [1, 2].

During a "seeding" phase, new daily infections are generated using a binomial probability distribution and the international air traffic data from the Australian Bureau of Infrastructure, Transport, and Regional Economics (BITRE). These infections are then assigned to agents residing in randomly selected areas within a certain radius from an airport [3]. For example, newly generated daily infections within Greater Sydney's boundaries are distributed within a 50 km radius of Sydney's international airport.

Agent states include: Susceptible, Latent, Infectious (asymptomatic or symptomatic), and Removed (recovered or dead). The set G_i contains all mixing contexts (e.g., residential, workplace, etc.) of agent i. At time step n, the infection probability for susceptible agent i across context $g \in G_i$ is determined as follows:

$$p_i^g(n) = 1 - \prod_{j \in A_g \setminus \{i\}} (1 - p_{j \to i}^g(n)) \tag{1}$$

where $A_g \setminus \{i\}$ is the list of agents in the context $g \in G_i$ excluding agent i, and $p_{j \to i}^g(n)$ is the instantaneous probability that an infectious agent j, sharing the context g with susceptible agent i, transmits the infection to agent i:

$$p_{j \to i}^g(n) = \kappa \; f(n - n_j | j) \; q_{j \to i}^g \tag{2}$$

A global transmission scalar κ calibrates the reproductive number R_0 (see sensitivity analysis in section Calibration and sensitivity analysis.). The step n_j marks the time when agent j becomes infected, and a function $f(n - n_j | j)$ represents the natural history of the disease, i.e., the infectivity of agent j over time. For an uninfected agent j, $n < n_j$ and $f(n - n_j | j) = 0$. For an infected agent j, $n \geq n_j$ and $f(n - n_j | j) \geq 0$. The infectivity increases exponentially until its peak, $f(n - n_j | j) = 1.0$. After the peak, during the recovery period, the infectivity decreases linearly to 0.0, when the agent state changes to Removed. The age- and context-dependent daily probabilities of transmission from agent j to agent i, denoted by $q_{j \to i}^g$, are specified in Table A in S1 Text, following prior studies [4, 5].

Asymptomatic agents are modeled to be less infectious than symptomatic agents, with their infectivity scaled down by factor α_{asymp}. The sensitivity of the model to changes in this factor is explored in section Calibration and sensitivity analysis.

The infection probability for agent i across all mixing contexts is determined as follows:

$$\begin{aligned} p_i(n) &= 1 - \prod_{g \in G_i(n)} (1 - p_i^g(n)) \\ &= 1 - \prod_{g \in G_i(n)} \prod_{j \in A_g \setminus \{i\}} \left(1 - p_{j \to i}^g(n)\right) \end{aligned} \tag{3}$$

At the end of each time step, the probability $p_i(n)$ is used by Bernoulli sampling to determine whether a susceptible agent i becomes infected.

Mixing context	Type of interaction	Daily transmission probability ($q^g_{j\to i}$)
Household (size 2)	Any to child (0 - 18)	0.09335
	Any to adult (19+)	0.02420
Household (size 3)	Any to child (0 - 18)	0.05847
	Any to adult (19+)	0.01495
Household (size 4)	Any to child (0 - 18)	0.04176
	Any to adult (19+)	0.01061
Household (size 5)	Any to child (0 - 18)	0.03211
	Any to adult (19+)	0.00813
Household (size 6)	Any to child (0 - 18)	0.02588
	Any to adult (19+)	0.00653
Household Cluster	Child (0 - 18) to child (0 - 18)	0.00400
	Child (0 - 18) to adult (19+)	0.00400
	Adult (19+) to child (0 - 18)	0.00400
	Adult (19+) to adult (19+)	0.00400
Working Group	Adult (19+) to adult (19+)	0.00400
School	Child (0 - 18) to child (0 - 18)	0.00029
Grade	Child (0 - 18) to child (0 - 18)	0.00158
Class	Child (0 - 18) to child (0 - 18)	0.00865
Neighborhood	Any to child (0 - 4)	0.035×10^{-5}
	Any to child (5 - 18)	1.044×10^{-5}
	Any to adult (19 - 64)	2.784×10^{-5}
	Any to adult (65+)	5.568×10^{-5}
Community	Any to child (0 - 4)	0.872×10^{-6}
	Any to child (5 - 18)	2.608×10^{-6}
	Any to adult (19 - 64)	6.960×10^{-6}
	Any to adult (65+)	13.92×10^{-6}

Table A. Daily transmission probabilities $q^g_{j\to i}$ from infected agent j to susceptible agent i for different mixing contexts and interaction types. Numbers in brackets show age groups.

The probability of symptomatic illness is then calculated by adjusting the infection probability $p_i(n)$ by a scaling factor σ representing the fraction of symptomatic cases over the total cases:

$$p_i^d(n) = \sigma(i)\ p_i(n) \tag{4}$$

The fraction $\sigma(i)$ is specified for adults (age ≥ 18), $\sigma_a = 0.67$, and children (age < 18), $\sigma_c = 0.268$, following prior studies [4], and is varied in sensitivity analysis, see section Calibration and sensitivity analysis. The main parameters of the ABM are summarised in Table B and Table C in S1 Text.

Non-pharmaceutical Interventions. The model includes several non-pharmaceutical interventions (NPIs): case isolation (CI), home quarantine (HQ), school closures (SC), and social distancing (SD). Each NPI is defined by (i) the population fraction that adopts it, and (ii) the adjusted (typically, decreased) strengths

Parameter	Value	Distribution	Notes
κ	23.0	constant	global transmission scalar
T_{inc}	3 days (mean)	lognormal ($\mu = 1.013,\ \sigma = 0.413$)	incubation period
T_{rec}	9 days (mean)	uniform [7, 11]	recovery period
α_{asymp}	0.3	constant	asymptomatic transmission scalar
σ_a	0.67	constant	probability of symptoms (age < 18)
σ_c	0.268	constant	probability of symptoms (age 18+)
π_{symp}	0.1	constant	daily case detection probability (symptomatic)
π_{asymp}	0.01	constant	daily case detection probability (asymptomatic)

Table B. Main input parameters for AMTraC-19 transmission model.

Parameter	Value and 95% CI	Sample size	Notes
R_0	19.56 [19.12, 19.65]	7,548	basic reproductive ratio
T_{gen}	5.42 [5.38, 5.44]	7,548	generation/serial interval

Table C. Derived epidemiological parameters.

of interactions between an NPI-adopting agent and other agents within their mixing groups, see Table D in S1 Text. The infection probability $p_i(n)$ for NPI-adopting agents is adjusted as follows:

$$p_i(n) = 1 - \prod_{g \in G_i(n)} \left[1 - F_g(i) \left(1 - \prod_{j \in A_g \setminus \{i\}} (1 - F_g(j)\ p^g_{j \to i}(n)) \right) \right] \quad (5)$$

where $F_g(j) \neq 1$ is the strength of the interaction between agent j and other agents in the mixing context g. For non-adopting agents j, the interaction strength is unchanged: $F_g(j) = 1$.

For each agent j adopting multiple NPIs, the value of $F_g(j)$ is preferentially assigned to only one NPI in accordance with the following order: CI, HQ, SD, SC. At each time step and for each NPI, the NPI-adopting and non-adopting agents are randomly selected according to Bernoulli process. The NPI-adoption fractions for CI, HQ, and SC are fixed during the simulation. The SD-adoption fraction, however, is chosen according to an optimised assignment profile, presented in Table E in S1 Text.

The resultant profile of SD-adoption is produced as a result of: (i) partitioning the simulated timeline with a number h of change-points, limited by the number of the modelled pandemic phases, $h < 6$; (ii) varying these change-points in increments of 5 days; (iii) varying the fractions within each partitioned period, in increments of 0.1 between $SD_{min} = 0$ and $SD_{max} = 0.7$, with fractions $SD_{max} > 0.7$ assumed to be infeasible.

Vaccination modelling We simulated a pre-emptive vaccination rollout which immunised 10.53M (45% of the population) with "priority" vaccine and 10.53M (45% of the population) with "general" vaccine, reaching 90% vaccination coverage nationwide. The coverage included 3.4M agents under 18 years of age ($age < 18$), 14.3M agents between 18 and 65 years of age ($18 \leq age < 65$), and 3.4M agents at or over 65 years of age ($age \geq 65$), see Table F in S1 Text. In each simulation, the agents were immunised prior to the start of the Omicron stage.

In setting vaccine efficacy levels, we followed study of Andrews et al. [6] which reported that after 2-4 weeks, the efficacy of boosted BNT162b2 (Pfizer/BioNTech) is

	Macro-distancing			Micro-distancing (interaction strengths)			
Intervention	Compliance level	Duration T	Threshold	Household	Community	Workplace \School	Duration t
CI	0.7	196	0	1.0	0.25	0.25	$D(i)$
HQ	0.5	196	0	2.0	0.25	0.25	7
SC^c	1.0	110	100	1.0	0.5	0	110
SC^a	0.25	110	100	1.0	0.5	0	110
Static SD	$[0.2, 0.7]$	196	400	1.0	0.25	0.1	196

Table D. The macro-distancing parameters and interaction strengths of NPIs in the studied scenarios. The micro-duration of CI is limited by the disease progression in the affected agent i, $D(i)$.

Dynamic profile (23.4M agents)		Dynamic profile (scaled to 25.8M agents)	
SD-adoption fraction	Simulation period (days)	SD-adoption fraction	Simulation period (days)
0.3	0-54	0.3	0-54
0.7	55-94	0.7	55-94
0.6	95-109	0.6	95-109
0.4	110-124	**0.5**	110-**129**
0.5	125-139	**0.6**	**130**-139
0.2	140-196	**0.3**	140-196

Table E. Macro-distancing dynamic SD-adoption fractions optimisation. (Left): approximately 23.4M population (2016 census), and (right): approximately 25.8M population (scaled by 10% relative to the 2016 census). The scaling-induced differences in best-fit SD-adoption fractions and simulation periods are highlighted in bold. Micro-distancing interaction strengths are the same as in Table D in S1 Text. Note that the initial SD-adoption of 0.3 is triggered when cumulative incidence reaches 400 around day 18 (specific days vary between different runs).

67.2% (95% CI, 66.5 to 67.8) and the efficacy of mRNA-1273 (Moderna) as 73.9% (95% CI, 73.1 to 74.6). For simplicity, we categorise these two vaccines as the "priority" vaccine with clinical efficacy set at $VE^c \approx 0.7$. Lower vaccine efficacy was reported among people who received ChAdOx1 nCoV-19 (Oxford/AstraZeneca), and we set $VE^c \approx 0.5$ for "general" vaccine. The clinical efficacy VE^c is further split into the efficacy for susceptibility (VE^s) and the efficacy for disease (VE^d), following prior studies [7]:

$$VE^c = VE^d + VE^s - VE^s \times VE^d \tag{6}$$

where $VE^d = VE^s = 0.452$ for priority vaccine, and $VE^d = VE^s = 0.293$ for general vaccine. Compared to the Delta variant, a lesser efficacy against transmission (VE^t) against Omicron has been reported [8], and we set $VE^t = 0.4$ for both types of vaccines considered in this study. A sensitivity analysis testing a range of VE^t and VE^c values was performed in prior studies [4, 7], showing that the model is robust to changes in the efficacy components.

For all vaccinated agents j we set $VE^t_j = VE^t$, $VE^s_j = VE^s$ and $VE^d_j = VE^d$, and for all unvaccinated agents $VE^t_j = VE^s_j = VE^d_j = 0$. The transmission probability of infecting a susceptible agent i is derived as follows:

$$p_i(n) = 1 - \prod_{g \in G_i(n)} \left[1 - (1 - VE^s_i) F_g(i) \left(1 - \prod_{j \in A_g \setminus i} (1 - (1 - VE^t_j) F_g(j)\ p^g_{j \to i}(n)) \right) \right] \tag{7}$$

The probability of becoming ill (symptomatic) is further affected by the efficacy against disease (VE^d_i): $p^d_i(n) = (1 - VE^d_i)\ \sigma_{a|c}\ p_i(n)$, given the fractions σ_a and σ_c of symptomatic adults and children, respectively.

Mortality statistics Actual daily and cumulative deaths are derived from the reported weekly mortality for both (i) COVID-19 deaths, and (ii) COVID-19 related deaths, see Figs 1, 5 and Fig A in S1 Text. This distinction differentiates between (i) doctor-certified deaths where COVID-19 is the underlying cause of death, and (ii) deaths where COVID-19 is both the underlying cause of death or a contributing factor (i.e., dying from or with COVID-19) [9].

During phases 6 and 7 of the Omicron pandemic stage, these two causes of death diverged more markedly, with the ratio between COVID-19 deaths and COVID-19 related deaths averaging to 0.77, see Fig B in S1 Text. This ratio may also be used, during phases 6 and 7, as a proxy to differentiate between the ICU occupancy resulting from or with COVID-19, as shown in Fig 4, where the corresponding simulated trajectories are adjusted for phase 6 by scaling with 0.77.

Modelling disease burden We modelled disease burden in terms of hospitalisations (occupancy), ICU cases (occupancy), and daily and cumulative deaths. For daily hospitalisations, we scaled the age-dependent case hospitalisation risks (CHRs) from the Alpha variant (B.1.1.7) reported by Nyberg et al. [10] to the Omicron variant (BA.1) by performing a linear regression between (i) the hospitalisation cases computed using CHRs for the Alpha variant, and (ii) the actual hospitalisation cases in Australia between 21 December 2021 and 15 January 2022. The regression shows a strong fit ($R^2 = 0.9921$) with the multiplier of 0.44 and the additive constant of 472.9 (Fig C and Table G in S1 Text). Hospital admissions were set to follow infections by 7 days, with the offset derived by aligning the first peak for simulated and actual trajectories, and the average hospital stay was assumed to be 6 days, in agreement with various

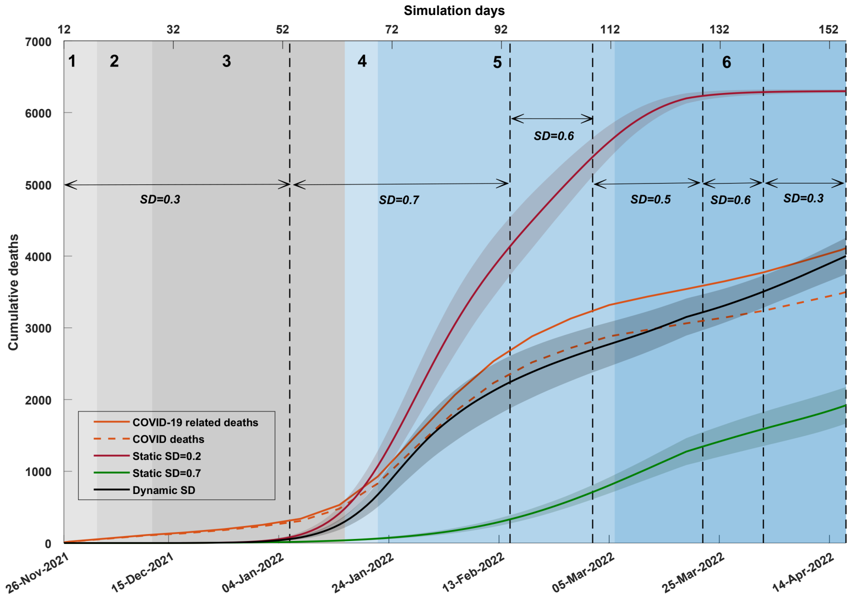

Fig A. A comparison of cumulative deaths produced by different social distancing (SD) profiles. The plot contrasts dynamic SD levels (solid black) and static SD levels ($SD_1 = 0.2$, shown in red; $SD_2 = 0.7$, shown in green). The simulated deaths are offset by 14 days. Coloured shaded areas around the solid line show standard deviation. Changes in dynamic SD-adoption are marked by vertical dashed black lines. Traces corresponding to each simulated scenario are computed as the average over 20 runs. SD adoption is combined with other interventions (i.e., school closures, case isolation, and home quarantine). Actual cumulative deaths are derived using reported weekly mortality (shown in orange; solid: COVID-19 related deaths; dashed: COVID-19 deaths). Shaded areas in grey and blue show the emergence of variants of concern and sub-lineages over time, identified in weekly genomic surveillance reports (NSW Health). The timeline is divided into 6 phases as follows: 1) BA.1 detected, 2) Delta and BA.1 co-exist, 3) BA.1 dominant, 4) BA.2 detected, 5) BA.1 and BA.2 co-exist, and 6) BA.2 dominant.

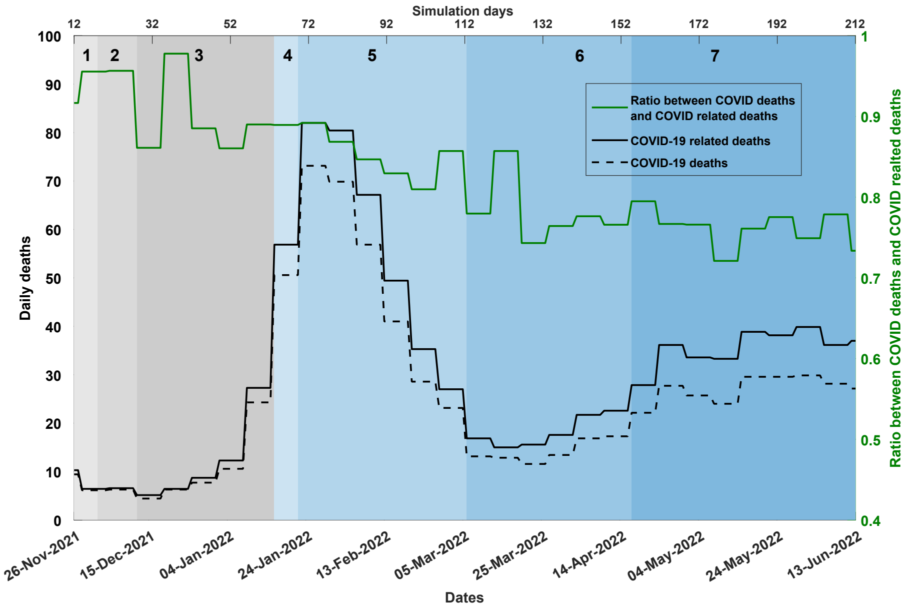

Fig B. COVID-19 mortality data for Australia between 26th November 2021 and 13th June 2022. Shaded areas in grey and blue show the emergence of variants of concern and sub-lineages over time, identified in weekly genomic surveillance reports (NSW Health). The timeline is divided into 7 phases as follows: 1) BA.1 detected, 2) Delta and BA.1 co-exist, 3) BA.1 dominant, 4) BA.2 detected, 5) BA.1 and BA.2 co-exist, 6) BA.2 dominant, and 7) BA.2, BA.4 and BA.5 co-exist. Solid black line (y-axis, left): COVID-19 related deaths; dashed black line (y-axis, left): COVID-19 deaths. Solid green line (y-axis, right): ratio between COVID-19 deaths and COVID-19 related deaths, with an average of 0.77 during phases 6 and 7.

Parameter	Value	Reference
Priority vaccine coverage	10.53M	45% of total population
General vaccine coverage	10.53M	45% of total population
Vaccine allocation [$age < 18$]	3.4M	16.1% of vaccinated population
Vaccine allocation [$18 \leq age \leq 65$]	14.3M	67.8% of vaccinated population
Vaccine allocation [$age \geq 65$]	3.4M	16.1% of vaccinated population
Priority, VE_d	0.7	[6]
Priority, VE_s	0.452	derived
Priority, VE_d	0.452	derived
Priority, VE_i	0.4	[8]
General, VE_d	0.5	[6]
General, VE_s	0.293	derived
General, VE_d	0.293	derived
General, VE_i	0.4	[8]

Table F. Parameters of pre-emptive vaccination rollout simulated with the ABM.

reports [11–13]. The risks of severe disease outcomes (i.e., hospitalisations, ICU admission and mortality) for vaccinated individuals are reduced by applying corresponding scalars summarised in Table G in S1 Text.

Daily ICU admissions were computed as fractions of the daily hospitalisations by applying age-dependent ratios. These ratios were approximated between the actual ICU occupancy and actual hospitalisations occupancy reported in NSW between 26 November 2021 and 12 February 2022 [14], summarised in Table G in S1 Text. ICU admissions were set to follow infections by 7 days, with the offset derived by aligning the first peak for simulated and actual trajectories, and the average ICU stay was assumed to be 5 days during the first five phases, and 4 days during phase 6 [11–13].

We then estimated the potential mortality, given the incidence cases, by scaling the age-dependent infection fatality rates (IFRs) for the Delta variant [4], reducing the risk of death for the Omicron variant to be 66% lower compared to the Delta variant [15]. Deaths were set to follow infections by 14 days, with the offset derived by aligning the first peak for simulated and actual trajectories.

In order to capture a reduction in the disease severity of BA.2 relative to BA.1, reported elsewhere [16, 17], we differentiated between sub-variants BA.1 and BA.2. The risks of hospitalisations, ICU admissions and mortality for both vaccinated and unvaccinated individuals are adjusted as follows:

$$\begin{aligned} \mathrm{CHR}_{\mathrm{BA.k}} &= \mathrm{CHR}_{\mathrm{Omicron}} \times V^{\mathrm{CHR}}_{\mathrm{BA.k}} \\ \mathrm{ICU}_{\mathrm{BA.k}} &= \mathrm{ICU}_{\mathrm{Omicron}} \times V^{\mathrm{ICU}}_{\mathrm{BA.k}} \\ \mathrm{IFR}_{\mathrm{BA.k}} &= \mathrm{IFR}_{\mathrm{Omicron}} \times V^{\mathrm{IFR}}_{\mathrm{BA.k}} \end{aligned} \tag{8}$$

where $k = 1$ or $k = 2$, and the default risks and rates for Omicron are presented in Table G in S1 Text.

The vaccine efficacy against the severe disease caused by the Omicron variant is

Rate % \ Age	0-9	10-19	20-29	30-39	40-49	50-59	60-69	70-79	80+
CHR_{Alpha}	0.9	0.7	1.9	3.4	5.0	7.2	10.6	16.9	21.7
$CHR_{Omicron}$	0.40	0.31	0.84	1.50	2.20	3.17	4.66	7.44	9.55
IFR_{Delta}	0.0012	0.0024	0.010	0.011	0.036	0.11	0.68	1.81	4.38
$IFR_{Omicron}$	0.0004	0.0008	0.0034	0.0037	0.0122	0.0374	0.2312	0.6154	1.4892
$ICU_{Omicron}$	0.034	0.066	0.052	0.075	0.12	0.15	0.17	0.14	0.06

Table G. Estimates of age-dependent case hospitalisation risks (CHRs, %), infection fatality rates (IFRs, %) and ICU admission rates. ICU admission rates are published in weekly COVID-19 surveillance reports prepared by NSW Health [14]. IFRs for Omicron are estimated to be 66% lower than the Delta variant [19].

Parameter	Value	Notes
Omicron CHR vaccination scalar, $V^{CHR}_{Omicron}$	0.25	[18]
Omicron ICU vaccination scalar, $V^{ICU}_{Omicron}$	0.60	derived
Omicron IFR vaccination scalar, $V^{IFR}_{Omicron}$	0.20	[18]
BA.1 CHR scalar, $V^{CHR}_{BA.1}$	1.0	[16]
BA.1 ICU scalar, $V^{ICU}_{BA.1}$	1.0	[16]
BA.1 IFR scalar, $V^{IFR}_{BA.1}$	1.0	[16]
BA.2 CHR scalar, $V^{CHR}_{BA.2}$	0.86	[16]
BA.2 ICU scalar, $V^{ICU}_{BA.2}$	0.85	[16]
BA.2 IFR scalar, $V^{IFR}_{BA.2}$	0.42	[16]

Table H. Vaccination scalars for the Omicron variant and BA.1/BA.2 differentiation.

assumed to be 75% [18]. This means that the age-dependent case hospitalisation risks for vaccinated individuals, $CHR^{vacc}_{BA.k}$, need to be further scaled down from the values $CHR_{BA.k}$ for both sub-variants BA.1 and BA.2, by Omicron CHR vaccination scalar $V^{CHR}_{Omicron} = 0.25$:

$$CHR^{vacc}_{BA.k} = CHR_{BA.k} \times V^{CHR}_{Omicron} \tag{9}$$

The age-dependent risks of ICU admission for vaccinated individuals, $ICU^{vacc}_{BA.k}$, are also reduced from the risks $ICU_{BA.k}$ for both sub-variants, by Omicron ICU vaccination scalar ($V^{ICU}_{Omicron}$):

$$ICU^{vacc}_{BA.k} = ICU_{BA.k} \times V^{ICU}_{Omicron} \tag{10}$$

We varied parameter $V^{ICU}_{Omicron}$ in a range [0.5, 0.7] with 0.1 increment, and found that $V^{ICU}_{Omicron} = 0.6$ provides the best fit to the actual ICU occupancy in Australia.

The efficacy against death caused by the Omicron variant is assumed to be 80% [18]. Thus, the age-dependent infection fatality rates for vaccinated individuals, $IFR^{vacc}_{BA.k}$, are accordingly adjusted by Omicron IFR vaccination scalar ($V^{IFR}_{Omicron} = 0.2$), reducing the values of $IFR_{BA.k}$ for both sub-variants:

$$IFR^{vacc}_{BA.k} = IFR_{BA.k} \times V^{IFR}_{Omicron} \tag{11}$$

Table H in S1 Text summarises different vaccination scalars used for adjusting risks and rates for the Omicron variant and BA.1/BA.2 differentiation.

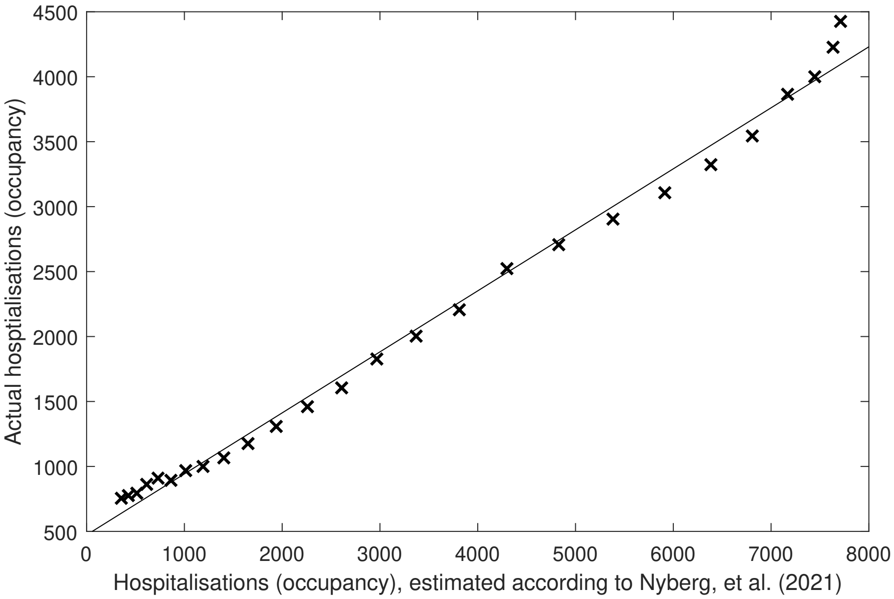

Fig C. Linear regression. Linear regression between case hospitalisation risk (CHRs) for the Alpha variant and the hospitalisation cases reported in Australia between between 21 December 2021 and 15 January 2022 produced a strong fit, with $R^2 = 0.9921$.

Calibration and sensitivity analysis We calibrated the model to transmission of the Omicron variant in Australia, focusing at sub-variant BA.1, but considering its mix with BA.2. By varying the global transmission scalar (κ), we explored a range of the reproduction number (R_0). Our aim was to attain R_0 at least 3.1 times higher than the reproduction number of the Delta variant [20]. The latter was calibrated in our prior studies, using similar ABMs, as being close to $R_0 = 6.20$ (with 95% CI of 6.16–6.23, N = 6,609; at the scale of Australia) [4], and $R_0 = 6.35$ (with 95% CI 5.86–6.84, $N = 300$; at the scale of New South Wales) [5].

To derive R_0 for the Omicron variant (as a mix of sub-variants BA.1 and BA.2), we carried out 7,548 simulations, each time randomly selecting an agent as the primary case, tracing transmissions, and counting only the direct secondary cases. We then used "the attack rate pattern weighted index case" method, eliminating a bias in selecting the primary case [21, 22]. The following age-specific attack rates were determined by the primary simulation: [0.0502, 0.1229, 0.1527, 0.4951, 0.1791], for five age groups [0-4, 5-18, 19-29, 30-64, 65+]. The process produced $R_0 = 19.56$ (with 95% CI 19.12–19.65, $N = 7,548$), see Table C in S1 Text.

We also varied the daily case detection probability (symptomatic) π_{symp}, in the range of 0.08 and 0.23, with $\pi_{symp} = 0.1$ producing the best fit to the actual incidence data. The parameters were calibrated to the Omicron stage in Australia between 26 November 2021 and 16 April 2022, as shown in Fig B in S1 Text and summarised in Table B in S1 Text.

We performed a local point-based sensitivity analysis to examine the robustness of the ABM, quantifying the changes in the peak incidences in response to changes in parameters of interest, while using default values for the other input parameters, see Table B in S1 Text. We varied the following three parameters: the global transmission scalar (κ), the fraction of symptomatic cases (σ_a), and the infectivity of asymptomatic cases (α_{asymp}). The changes in these parameters were evaluated with respect to two

	First incidence peak				Second incidence peak			
κ	mean	median	25% quantile	75% quantile	mean	median	25% quantile	75% quantile
21 ($R_0 = 17.81$)	70,287	69,859	61,052	79,136	82,719	79,371	69,714	91,127
22 ($R_0 = 18.70$)	79,781	80,025	67,438	90577	74,069	75,115	65,097	81,663
**23 ($R_0 = 19.56$)**	**100,030**	**104,210**	**88,537**	**113,110**	**64,033**	**63,061**	**57,124**	**69,735**
24 ($R_0 = 20.14$)	116,890	117,770	99,451	129,660	57,488	57,833	54,070	61,579
25 ($R_0 = 20.75$)	142,320	142,760	134,660	151,950	54,462	53,826	47,149	58,650

Table I. Statistics of the local sensitivity analysis tracing incidence peaks with respect to changes in the global transmission scalar (κ) over 20 runs. The default value is in bold.

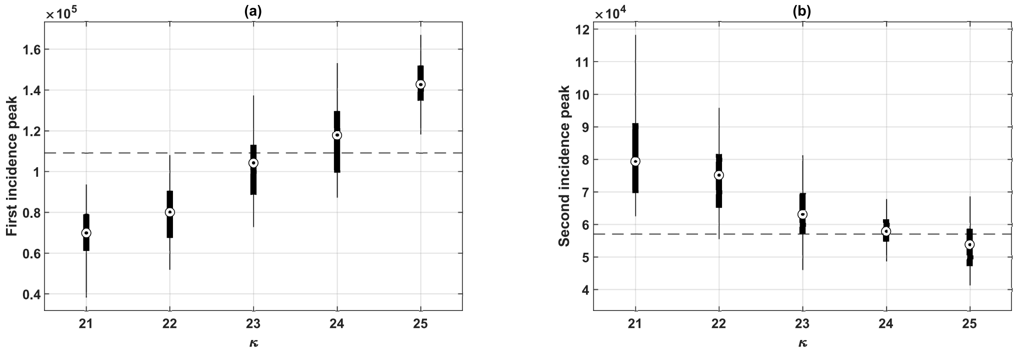

Fig D. Local sensitivity analysis tracing incidence peaks with respect to changes in the global transmission scalar (κ) over 20 runs. The black dashed line marks the actual incidence peak. (a) First incidence peak (actual 7-day moving average incidence peak in early January). (b) Second incidence peak (actual 7-day moving average incidence peak in early April).

incidence peaks (global maximum in mid-January, 2022; and local maximum in mid-April, 2022), shown in Fig D to Fig F in S1 Text.

Global transmission scalar. We varied the global transmission scalar (κ) in the range between 21 and 25, with the increment step of 1, centred around the default value at 23, simulated under the dynamic SD scenario. Other intervention conditions and parameters were unchanged. Fig D and Table I in S1 Text show the corresponding changes in the two incidence peaks.

In summary, higher κ results in higher first peak and lower second peak. This is expected, since κ directly scales the reproduction number R_0 from 17.81 to 20.75, as shown in Table I in S1 Text. In close proximity to the default value $\kappa = 23$, the robustness of the model is not challenged by showing linear dependencies between the incidence peaks and κ.

Asymptomatic infectivity. We model asymptomatic individuals with lower infectivity compared to their symptomatic counterparts. The asymptomatic infectivity is governed by asymptomatic infectivity α_{asymp} varying between 0 and 1, interpreted as the relative infectivity of an asymptomatic individual compared to the maximum level of an symptomatic case. We varied α_{asymp} within the range $[0.1, 0.5]$ with an increment step of 0.1. Fig E and Table J in S1 Text show the corresponding changes in two incidence peaks.

Higher asymptomatic infectivity produces higher first incidence peak and lower second incidence peak. At the lower bound $\alpha_{asymp} = 0.1$, the first peak incidence has mean value of $8,892$ and increases to $171,770$ at the higher bound $\alpha_{asymp} = 0.5$. The decrease in second incidence, on the other hand, is less sensitive to the change of α_{asymp}. The default setting $\alpha_{asymp} = 0.3$ is in concordance with the actual incidence

	First incidence peak				Second incidence peak			
α_{asymp}	mean	median	25% quantile	75% quantile	mean	median	25% quantile	75% quantile
0.1	8,892	8,403	7,356	9,989	141,470	140,790	135,290	146,450
0.2	44,296	41,952	40,036	48,736	111,090	108,940	103,010	120,390
**0.3**	**100,030**	**104,210**	**88,537**	**113,110**	**64,033**	**63,061**	**57,124**	**69,735**
0.4	151,870	159,190	139,620	170,920	48,993	49,769	43,323	54,205
0.5	171,170	179,040	156,550	188,170	45,971	44,908	36,530	52,599

Table J. Statistics of the local sensitivity analysis tracing incidence peaks with respect to changes in the infectivity of asymptomatic cases (α_{asymp}), over 20 runs. The default value is in bold.

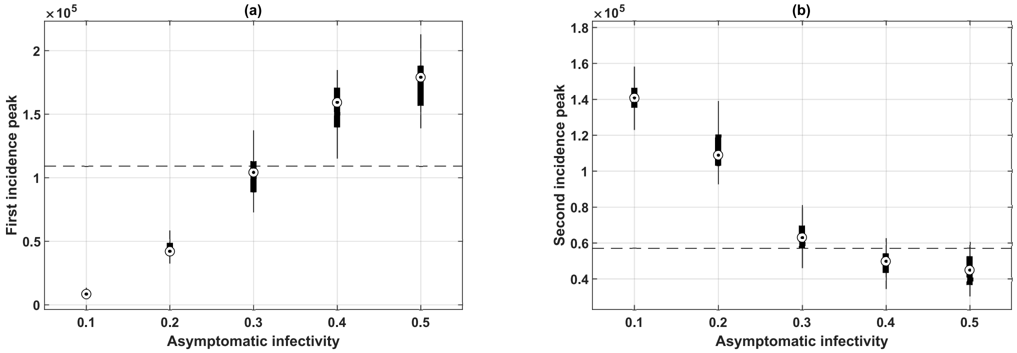

Fig E. Local sensitivity analysis tracing incidence peaks with respect to changes in the infectivity of asymptomatic cases (α_{asymp}) over 20 runs. The black dashed line marks the actual incidence peak. (a) First incidence peak (actual 7-day moving average incidence peak in early January). (b) Second incidence peak (actual 7-day moving average incidence peak in early April).

peaks (marked by black dashed line).

Symptomatic fraction (adults). Our model splits infected adults into symptomatic and asymptomatic cases, governed by the symptomatic fraction (adults), σ_a. This input parameter was tested within the range $[0.47, 0.87]$ with an incremental step of 0.1, centred around the default value of 0.67. Fig F and Table K in S1 Text summarise the changes in incidence peaks.

As σ_a increases, the first incidence peak is more responsive to parameter change, increasing from 57,272 (mean) at the lower bound at $\sigma_a = 0.47$ to 151,550 (mean) at the higher bound $\sigma_a = 0.87$, while the second incidence shows low sensitivity with the peak reducing from 74,743 (mean) to 66,272 (mean).

Summary. We performed local sensitivity analysis by varying three key input

	First incidence peak				Second incidence peak			
σ_a	mean	median	25% quantile	75% quantile	mean	median	25% quantile	75% quantile
0.47	57,272	56,841	47,895	64,190	74,743	74,027	65,218	82,092
0.57	76,324	77,454	64,723	86,298	68,156	66,868	56,221	75,436
**0.67**	**100,030**	**104,210**	**88,537**	**113,110**	**64,033**	**63,061**	**57,124**	**69,735**
0.77	126,540	132,270	109,160	143,740	61,268	61,795	54,413	68,199
0.87	151,550	156,640	117,320	180,980	66,272	62,589	60,710	70,509

Table K. Statistics of the local sensitivity analysis tracing incidence peaks with respect to changes in the fraction of symptomatic cases (σ_a), over 20 runs. The default value is in bold.

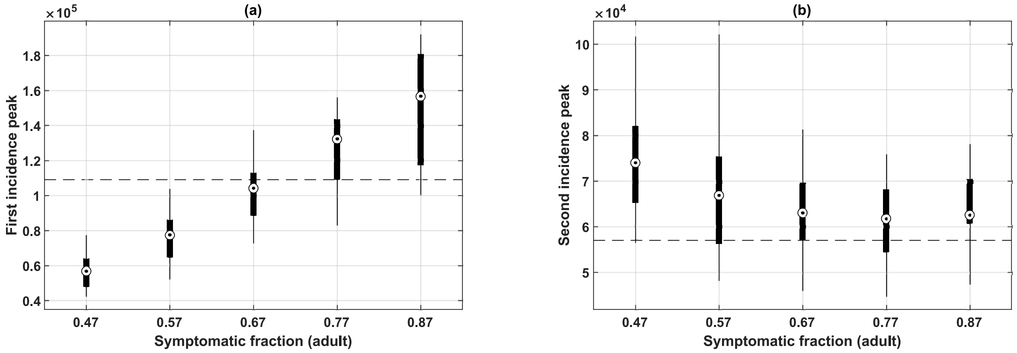

Fig F. Local sensitivity analysis tracing incidence peaks with respect to changes in the fraction of symptomatic cases (σ_a) over 20 runs. The black dashed line marks the actual incidence peak. (a) First incidence peak (actual 7-day moving average incidence peak in early January). (b) Second incidence peak (actual 7-day moving average incidence peak in early April).

Category	26/Nov/21 - 18/Dec/21<br>SD=0.3	07/Jan/22 - 02/Mar/22<br>SD= 0.7 and 0.6	03/Mar/22 - 02/Apr/22<br>SD=0.5 and 0.6	03/Apr/22 - 16/Apr/22<br>SD=0.3
Workplace (%)	-6.96	-17.07	-10.06	-12.67
Transport (%)	-38.50	-47.58	-40.77	-37.17

Table L. Mean mobility reduction (%) compared to the baseline. The reduction during a period between 26 November 2021 and 16 April 2022 in Australia, at workplace and transport stations, is compared to the baseline (i.e., the median value from the pre-pandemic 5-week period between 3 January to 6 February 2020) [23]. The mean mobility is shown in Fig G in S1 Text. Public holidays (e.g., Christmas and New Year break, Australia Day and Easter break) are excluded.

parameters (while keeping the remaining parameters at their default values in dynamic SD scenario, using the population of 23.4M agents and the corresponding SD assignment shown in Table E in S1 Text (left two columns with 23.4M agents): global transmission scalar (κ), the fraction of symptomatic cases (σ_a), and the infectivity of asymptomatic cases (α_{asymp}). We then tracked the changes in two incidence peaks, in order to measure the impact of these parameters. We observed that while both incidence peaks are sensitive to changes in infectivity of asymptomatic cases, the first incidence peak tends to be more sensitive to parameter change than the second incidence peak. Nevertheless, the model produces robust results for the parameter values in close proximity to the default values, which are in agreement with the actual incidence peaks.

Google mobility data We use Google COVID-19 mobility reports between 26 November 2021 and 16 April 2022 [23] to explore qualitative agreement between mobility trends and the "retrodictive" SD levels and corresponding periods. Here, we use mobility trends observed in workplace and transit stations (i.e., transport) as a secondary proxy because these two categories are more likely to have significantly reduced social interactions during SD periods. We find satisfactory agreement between the "retrodictive" SD periods and Google mobility reports by observing greater reduction in mobility during the period with high SD (e.g., higher reduction during periods with $SD = 0.7$ and $SD = 0.6$ than periods with $SD = 0.4$ and $SD = 0.5$, as shown in Fig G and Table L in S1 Text.

Comparison between dynamic and static SD-adoption Table M and Table N in S1 Text summarise data for the two incidence peaks during the Omicron stage of

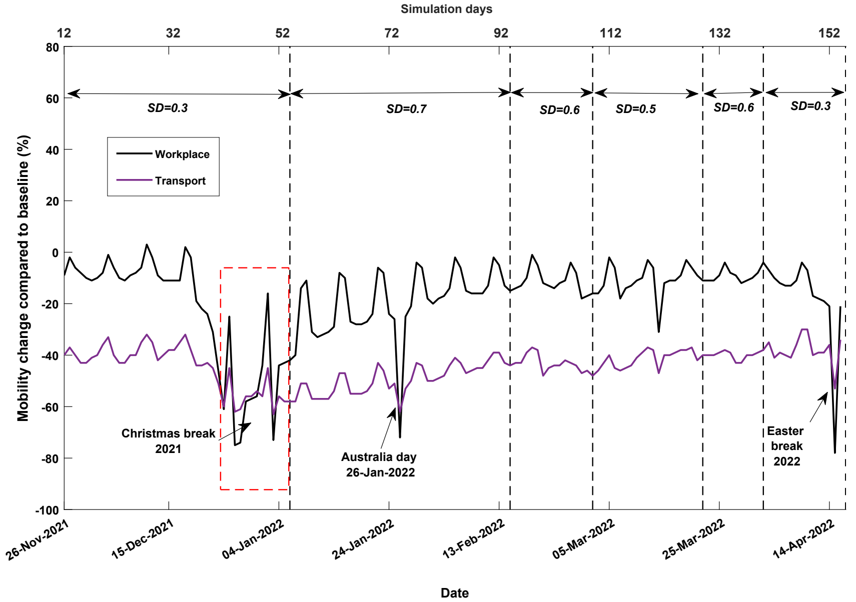

Fig G. Movement trends compared to the baseline. Movement trends between 26 November 2021 to 16 April 2022 in Australia at workplace and transport stations are compared to the baseline (i.e., the median value from the 5-week period between 3 January to 6 February 2020) [23].The dashed black lines mark the "retrodictively" estimated SD periods corresponding to the simulation results shown in Fig 2.

the pandemic in Australia. Three simulated scenarios (dynamic SD-adoption and two static SD-adoption alternatives) are contrasted with the actual data, in terms of daily incidence, hospitalisations, ICU occupancy and mortality.

SD-adoption	1st peak and STD	simulation day	2nd peak and STD	simulation day
0.2	185,000 (25,181)	65 (6)	None	None
0.7	41,000 (8,387)	108 (7)	56,000 (9,417)	141 (20)
Dynamic SD	104,000 (24,136)	60 (2)	69,000 (12,935)	136 (4)
Actual	109,100	10 January 2022	57,020	2 April 2022

Table M. Mean and standard deviation (STD) of incidence peaks produced by different social distancing (SD) profiles. We contrast static SD level of 0.2 and 0.7, and dynamic SD levels specified in S1 Table E. Incidence peak magnitude (daily cases) is averaged over 20 runs rounded to the nearest '000. The lower static SD-adoption, $SD_1 = 0.2$, fails to produce the second peak between 26 November 2021 to 16 April 2022.

SD-adoption	1st peak hospitalisations	1st peak ICU	2nd peak hospitalisations	2nd peak ICU	Cumulative deaths
0.2	8,700 (1,075)	600 (70)	None	None	6,300 (29)
0.7	2,100 (343)	210 (22)	None	None	2,000 (214)
Dynamic SD	5,200 (1,015)	370 (66)	3000 (453)	240 (29)	4,200 (116)
Actual	5,371	424	3,147	Not applicable	4,083

Table N. Mean and standard deviation (STD) of peak estimates of disease burden. Disease burden is measured in terms of the hospitalisations and ICU occupancy, and cumulative deaths (16 April 2022) in three SD scenarios: static SD level of 0.2 and 0.7, and dynamic SD levels specified in Table E in S1 Text. Peak magnitudes are averaged over 20 runs rounded to the nearest '00. The static SD-adoption fractions, $SD_1 = 0.2$ and $SD_2 = 0.7$, fail to produce the second peak within the considered timeline between 26 Nov 2021 to 14 Apr 2022.

References

1. Chang SL, Nguyen QD, Zachreson C, Cliff OM, Prokopenko M. AMTraC-19 Source Code: Agent-based Model of Transmission and Control of the COVID-19 pandemic in Australia; 2022. `https://doi.org/10.5281/zenodo.7325675`.

2. Cliff OM, Zachreson C, Harding N, Fair K, Piraveenan M, Prokopenko M. ACEMod (v19_12_2019) Source Code: Australian Census-based Epidemic Model; 2021. `https://zenodo.org/record/5773908`.

3. Cliff OM, Harding M, Piraveen M, Erten Y, Gambhir M, Prokopenko M. Investigating spatiotemporal dynamics and synchrony of influenza epidemics in Australia: an agent-based modelling approach. Simulation Modelling Practice and Theory. 2018;87:412–431.

4. Chang S, Cliff O, Zacherson O C Cliff, Prokopenko M. Simulating transmission scenarios of the Delta variant of SARS-CoV-2 in Australia. Frontiers in Public Health. 2022;10(823043).

5. Nguyen QD, Prokopenko M. A general framework for optimising cost-effectiveness of pandemic response under partial intervention measures. Scientific Reports. 2022;12:19482.

6. Andrews N, Stowe J, Kirsebom F, Toffa S, Rickeard T, Gallagher E, et al. Covid-19 vaccine effectiveness against the Omicron (B.1.1.529) variant. New England Journal of Medicine. 2022;386(16):1532–1546.

7. Zachreson C, Chang SL, Cliff OM, Prokopenko M. How will mass-vaccination change COVID-19 lockdown requirements in Australia? The Lancet Regional Health – Western Pacific. 2021;14:100224.

8. Buchan SA, Chung H, Brown KA, Austin PC, Fell DB, Gubbay JB, et al. Estimated Effectiveness of COVID-19 Vaccines Against Omicron or Delta Symptomatic Infection and Severe Outcomes. JAMA Network Open. 2022;5(9):e2232760–e2232760.

9. Australian Bureau of Statistics. Provisional Mortality Statistics. Provisional deaths data for measuring changes in patterns of mortality. Reference period: Jan - Jun 2022; 2022. `https://www.abs.gov.au/statistics/health/causes-death/provisional-mortality-statistics/latest-release`.

10. Nyberg T, Twohig KA, Harris RJ, Seaman SR, Flannagan J, Allen H, et al. Risk of hospital admission for patients with SARS-CoV-2 variant B.1.1.7: cohort analysis. BMJ. 2021;373.

11. Jassat W, Abdool Karim SS, Mudara C, Welch R, Ozougwu L, Groome MJ, et al. Clinical severity of COVID-19 in patients admitted to hospital during the Omicron wave in South Africa: a retrospective observational study. The Lancet Global Health. 2022;10(7):e961–e969.

12. Iuliano AD, Brunkard JM, Boehmer TK, Peterson E, Adjei S, Binder AM, et al. Trends in disease severity and health care utilization during the early Omicron variant period compared with previous SARS-CoV-2 high transmission periods – United States, December 2020 – January 2022. Morbidity and Mortality Weekly Report. 2022;71(4):146–152.

13. Tobin RJ, Wood JG, Jayasundara D, Sara G, Walker J, Martin G, et al. Hospital length of stay in a mixed Omicron and Delta epidemic in New South Wales, Australia. medRxiv. 2022;.

14. Department of Health, NSW. COVID-19 weekly surveillance reports - Archive; 2022. `https://www.health.nsw.gov.au/Infectious/covid-19/Pages/weekly-reports-archive.aspx`.

15. Lorenzo-Redondo R, Ozer EA, Hultquist JF. COVID-19: is Omicron less lethal than Delta? BMJ. 2022;378(o1806).

16. Sievers C, Zacher B, Ullrich A, Huska M, Fuchs S, Buda S, et al. SARS-CoV-2 Omicron variants BA.1 and BA.2 both show similarly reduced disease severity of COVID-19 compared to Delta, Germany, 2021 to 2022. Eurosurveillance. 2022;27(22).

17. Wolter N, Jassat W, author group DG, von Gottberg A, Cohen C. Clinical severity of Omicron lineage BA.2 infection compared with BA.1 infection in South Africa. The Lancet. 2022;400(10346):93–96.

18. Nyberg T, Ferguson NM, Nash SG, Webster HH, Flaxman S, Andrews N, et al. Comparative analysis of the risks of hospitalisation and death associated with SARS-CoV-2 Omicron (B.1.1.529) and Delta (B.1.617.2) variants in England: a cohort study. The Lancet. 2022;399(10332):1303–1312.

19. US Department of Health and Human Services/Centers for Disease Control and Prevention. Trends in disease severity and health care utilization during the early Omicron variant period compared with previous SARS-CoV-2 high transmission periods — United States, December 2020–January 2022; 2022. `https://www.cdc.gov/mmwr/volumes/71/wr/pdfs/mm7104e4-h.pdf`.

20. Obermeyer F, Jankowiak M, Barkas N, Schaffner SF, Pyle JD, Yurkovetskiy L, et al. Analysis of 6.4 million SARS-CoV-2 genomes identifies mutations associated with fitness. Science. 2022;376(6599):1327–1332.

21. Germann TC, Kadau K, Longini IM, Macken CA. Mitigation strategies for pandemic influenza in the United States. Proceedings of the National Academy of Sciences. 2006;103(15):5935–5940.

22. Zachreson C, Fair KM, Harding N, Prokopenko M. Interfering with influenza: nonlinear coupling of reactive and static mitigation strategies. Journal of The Royal Society Interface. 2020;17(165):20190728.

23. Google. COVID-19 Community Mobility Reports; 2022. https://www.google.com/covid19/mobility/.
